# The interaction between herpes simplex virus 1 genome and promyelocytic leukemia nuclear bodies (PML-NBs) as a hallmark of the entry in latency

**DOI:** 10.15698/mic2016.11.541

**Published:** 2016-11-04

**Authors:** Patrick Lomonte

**Affiliations:** 1 Univ Lyon, Université Claude Bernard Lyon 1, CNRS UMR 5310, INSERM U 1217, LabEx, DEVweCAN, Institut NeuroMyoGène (INMG), team Chromatin Assembly, Nuclear Domains, Virus; F-69100, Lyon, France.

**Keywords:** Herpesvirus, herpes simplex virus 1 (HSV-1), latency, neurons, nuclear architecture, promyelocytic leukemia nuclear bodies (PML-NBs), intrinsic immunity

## Abstract

Herpes simplex virus 1 (HSV-1) is a human pathogen that establishes latency in the nucleus of infected neurons in the PNS and the CNS. At the transcriptional level latency is characterized by a *quasi*-complete silencing of the extrachromosomal viral genome that otherwise expresses more than 80 genes during the lytic cycle. In neurons, latency is anticipated to be the default transcriptional program; however, limited information exists on the molecular mechanisms that force the virus to enter the latent state. Our recent study demonstrates that the interaction of the viral genomes with the nuclear architecture and specifically the promyelocytic leukemia nuclear bodies (PML-NBs) is a major determinant for the entry of HSV-1 into latency (Maroui MA, Callé A *et al*. (2016). Latency entry of herpes simplex virus 1 is determined by the interaction of its genome with the nuclear environment. PLoS Pathogens 12(9): e1005834.).

Nuclear replicating viruses have to face multiple layers of transcriptional controls following the entry of their genomes in the nucleus, regardless of integration into the host chromatin. Viral or proviral genome DNA modifications, assembly in chromatin, association with post-translationally modified canonical histones or histone variants, and positioning within the nuclear environment are epigenetic regulation features that positively or negatively influence the fate of the viral infection. After primary infection, herpesviruses establish latency in the infected host. Herpes simplex virus 1 (HSV-1) is a human neurotropic virus that remains in a latent state in neurons of the PNS and CNS with trigeminal ganglia (TG, also called Gasserian Ganglia) being the major sites triggered for virus latency.

The HSV-1 genome in the virion is a linear, naked, double-stranded DNA of about 150 kb programmed to encode more than 80 proteins. Two transcriptional programs are associated with the HSV-1 infection depending on whether the virus undergoes a lytic or a latent cycle. During the former the HSV-1 genome is fully transcribed, whereas the latter is characterized by a *quasi*-complete transcriptional silencing with the exception of the abundant expression of a family of non-coding RNAs called Latency Associated Transcripts (LATs). Once the viral genome is injected into the nucleus of the infected neuron, it circularizes, associates with nucleosomes, and remains as an episome, unintegrated in the host cell genome. This chromatinization of the viral genome is a hallmark of the latency process. The regulation of transcriptionally active and repressed promoters during latency has been shown to match to a certain extent with their association to nucleosomes that show similar histone post-translational modifications as found in euchromatin and heterochromatin, respectively. However, some discrepancies in the data regarding virus strains, route of infection in mouse models, mice genetic background, and the analyzed site of latency (e.g., TG versus dorsal root ganglia), suggest that latency, from a molecular and epigenetic point of view, is probably not homogenous in the whole infected tissue, and within individual neurons. This is probably an important aspect of the virus biology to take into consideration, especially for the development of future therapies designed to prevent virus reactivation from latency, and the associated pathologies.

To tackle this question we developed a combinatory fluorescence *in situ* hybridization (FISH)/immuno-fluorescence approach, specifically designed to visualize the viral genomes within individual infected neurons. Furthermore, we employed a mouse lip model of HSV-1 infection reproducing the latent infection of TGs. This strategy enabled us to analyze the overall nuclear distribution of HSV-1 genomes and their interaction with the nuclear environment from the initial stages of the viral genome entry into the nucleus of an infected neuron to the later stages, corresponding to latency. We concentrated our effort on the interaction of HSV-1 genomes with the promyelocytic leukemia (PML) nuclear bodies (NBs) because we knew from previous studies from our and other laboratories that HSV-1 genomes showed a particular affinity for PML-NBs.

PML-NBs are implicated in multiple cell processes, such as apoptosis, senescence and aging, but also act as nuclear sensors of multiple stresses including viral infections. Indeed, PML-NBs have been shown to negatively regulate the infection by DNA and RNA viruses. Our data showed that during the initial stage of infection of TG neurons (around 4 to 6 days post infection (dpi) in mice, also called acute infection), viral genome patterns specific for the lytic or latent transcriptional programs were present in individual neurons. The lytic program correlated with the presence of viral genome replication compartments (RC) in the nuclei of the infected neurons, whereas the latent program was characterized by multiple foci of viral genomes (up to 10 per infected neuron) scattered all-over the nucleoplasm; a phenotype we termed “multiple-acute” (MA). RC-containing neurons were systematically negative for the presence of PML-NBs. MA patterns were characterized by the co-localization of each viral genome foci with a PML-NB, with the PML protein forming a shell around the viral genomes. PML partners, such as the histone chaperones Daxx and ATRX, were also present within the structures. Because of the unusual presence of a DNA inside a PML-NB we proposed naming those structures “viral DNA-containing PML-NBs” (vDCP-NBs).

As infection time progressed, the RC and MA patterns progressively disappeared and two other viral genome patterns were visualized and remained until latency (28 dpi). vDCP-NBs were still visible in about half of the latently infected neurons, but this time only one vDCP-NB was detectable per infected neuron. We called this pattern “single” (S). *In vivo* observations and *in vitro* experiments suggested that the S pattern could result from the fusion of the multiple vDCP-NBs present in the MA pattern, which confers unexpected dynamics to these structures. The other latently infected neurons showed a distribution of viral genomes reminiscent of the MA seen during the acute phase but usually with a lot more viral genome foci per nuclei that, in general, did not co-localize with PML-NBs. We called this pattern “multiple-latency” (ML). We previously showed that only ML-containing neurons did express LATs.

These data are summarized in Figure 1. Under the ML pattern, the viral genomes were scattered in the nucleoplasm, and we showed in a previous study that a subset of them specifically co-localized with centromeres. Overall, these data suggested a strong correlation between the initial presence of viral genomes in vDCP-NBs and the establishment of latency in a subset of neurons.

**Figure 1:**
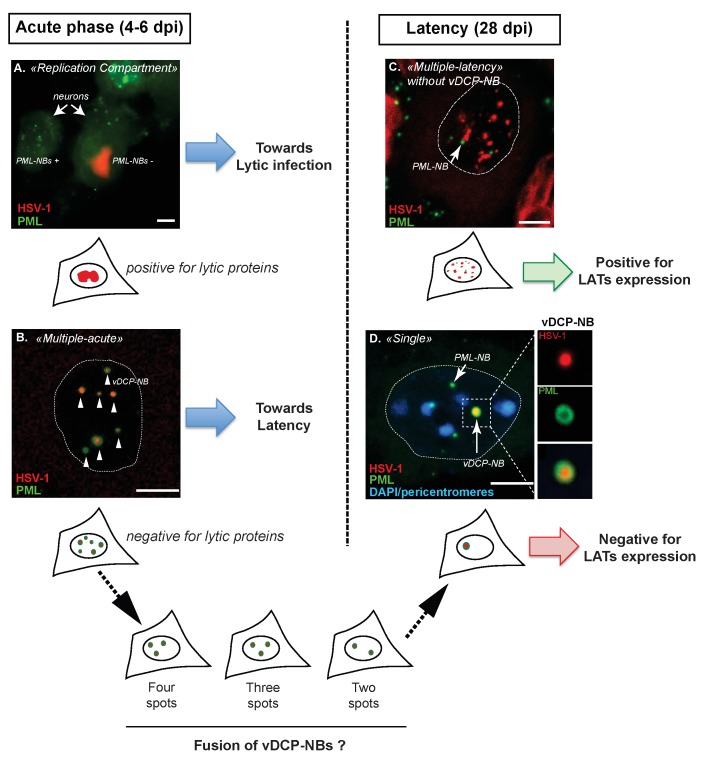
FIGURE 1: Major patterns of herpes simplex virus (HSV-1) genome distribution in infected trigeminal ganglion (TG) neurons during acute infection (4-6 dpi) and latency (28 dpi) in mice. HSV-1 genomes (red) are detected by fluorescence *in situ* hybridization (FISH) together with the detection of the PML protein (green) by immunofluorescence. During acute infection virus genomes adopt two main patterns: replication compartments (RC) or multiple-acute (MA). **(a)** RC are visualized by the detection of a “cloud” of viral genomes spread within the nucleoplasm. In RC-containing neurons, PML-NBs are no longer detected (compare infected right neuron with uninfected left neuron) and expression of lytic proteins could be detected. **(b)** MA is characterized by the detection of discrete spots of viral genomes surrounded by the PML protein. These structures were defined as “viral DNA-containing PML-NBs” (vDCP-NBs). No lytic proteins were detected in MA-containing neurons. During latency (from 28 dpi onwards), two other viral genome patterns are detectable: multiple-latency (ML) or single (S). **(c)** ML is distinguishable from MA based on at least three criteria: (i) the viral genome spots are usually smaller; (ii) the viral genome spots detected in the nucleus are more numerous; and (iii) most of the time ML-containing neuron do not contain any vDCP-NB. **(d) **S corresponds to the detection of only one spot of viral genome in the infected neuron, forming a unique vDCP-NB. The S pattern could result from the fusion of the multiple vDCP-NBs observed in MA-containing neurons during the period of latency establishment (between 6 and 28 dpi), since *in vivo*, intermediate patterns of 4-3-2 vDCP-NBs-containing neurons were observed around 11 to 14 dpi. Cartoons are present below each image for a general representation of the pattern. Blue colour in **(d)** represents DAPI staining. Dpi, days post infection. Bars represent 10 µm. Figure 1D was partly reproduced from a previous article by the author (Lomonte P. Virologie 2014; 18(3): 170-9. doi:10.1684/vir.2014.0569) who have obtained the agreement of the publisher to use it in the present publication.

In order to determine the viral and cellular features responsible for the acquisition of the latency-associated viral genome patterns, we infected cultured primary mouse TG neurons prepared from wild type or type I interferon (IFN) receptor knock out mice with a variety of mutant viruses. We found that the vDCP-NBs formation was favored in a context where the virus was unable to start its lytic program due to the combined absence of two of its major transactivator proteins, i.e., ICP4 and ICP0. Importantly, we found that viral genomes entrapped in the vDCP-NBs were not definitively silenced and could resume transcription, provided that the neurons were stressed by an appropriate stimulus. Moreover, the acquisition of the ML pattern was the consequence of the infection by a virus, which was able to start a lytic program but simultaneously under the pressure of the IFN-mediated antiviral response. Finally, immuno-FISH analyses of TG harvested from HSV-1 latently infected humans enabled the detection of vDCP-NB-like structures in latently infected neurons.

In summary, our study deciphered a major role of the PML-NBs in the antiviral response against the infection by a nuclear replicating virus. The interaction between HSV-1 genomes and PML-NBs forces the virus to enter a seemingly dormant state before the intervention of any IFN-associated antiviral mechanism. This highlights the importance of PML-NBs as restriction factors acting as nuclear relays of the intrinsic antiviral defense mechanism. The involvement of PML-NBs in sensing and silencing incoming viral genomes is likely a general mechanism that could control the infection by many viruses, whose infectious cycle occurs entirely or partly in the nucleus. Another important aspect of the study is the discovery that the fate of the infection by a nuclear replicating virus could be directly dependent on the interaction of its genome with the nuclear architecture. In addition, the mouse model teaches us that HSV-1 latency is heterogeneous from the point of view of viral genome nuclear distribution within individual infected neuron.

What could be the consequences of such diversity for the virus biology? First, it is worth considering that the transcriptional activity of a genome locus could be epigenetically regulated through its positioning inside the nucleus and its interaction with the microenvironment. Hence, it is tempting to speculate that latent viruses, such as HSV-1, by adopting various genome patterns (probably as a response to various cellular constrains some of which dependent on the IFN response) have evolved to increase their chances to achieve successful reactivations following various types of stresses to insure their propagation from host to host, and throughout multiple generations.

